# Awareness, utilization and influencing factors of social supports for main informal caregivers of schizophrenia patients: a cross-sectional study in primary care settings in Beijing, China

**DOI:** 10.1186/s12875-020-01257-z

**Published:** 2020-09-17

**Authors:** Meirong Wang, Guanghui Jin, Yun Wei, Feiyue Wang, Zhaolu Pan, Lifen Chen, Xiaoqin Lu

**Affiliations:** 1 School of General Practice and Continuing Education, Capital Medical University, Fengtai district, No. 10, You An Men Wai Xi Tou Tiao, Beijing, 100069 China; 2 Xuan Wu Hospital of Capital Medical University, Beijing, China

**Keywords:** Schizophrenia, Main informal caregiver, Mixed-methods approach, Beijing

## Abstract

**Background:**

Most schizophrenia patients are supported by main informal caregivers at home in China. This study aims to investigate the further needs of social supports for main informal caregivers of schizophrenia patients and to analyze influencing factors on the awareness and utilization of social supports in Beijing. The results of this study could potentially act as reference for health professionals to implement appropriate and effective support programs.

**Methods:**

A mixed-methods approach was used in this study. Awareness, utilization and influencing factors of social supports for main informal caregivers were investigated using questionnaires in 6 urban districts in Beijing. Meanwhile, individual in-depth interviews with 10 main informal caregivers from the urban districts of Beijing were conducted to identify the caregivers’ perspective on social supports and their further needs.

**Results:**

Quantitative results showed that although the government provided multi-channel resources for schizophrenia patients and their families, awareness and utilization of the resources such as rehabilitation and relevant subsidies were less than 10.0 and 5.0% respectively. Most caregivers in in-depth interviews expressed that they had negative experiences with respect to obtaining social supports, and they emphasized that more support would be needed in terms of financial support, respect, and rehabilitation institutions.

**Conclusions:**

The awareness and utilization of social supports are low for main informal caregivers of schizophrenia patients. More services and improved public attitudes are needed for schizophrenia patients and their caregivers.

## Background

Schizophrenia is a mental disorder characterized by hallucinations (visual, auditory, olfactory, or tactile) and delusions [1]. As a progressive disease, schizophrenia not only increases the risk of disability and dependency, but also affects the patients’ main informal caregivers’ lives. Globally, the population of schizophrenia rose from 13.1 million in 1990 to 20.9 million cases in 2016 [2]. China has a large schizophrenia population in the world, the pooled prevalence of schizophrenia from 1990 to 2016 was 0.42% [2]. There were approximately 16 million patients with mental illness in 2019 in China, of which schizophrenia patients accounted for about half, and the incidence of schizophrenia in urban areas was significantly higher than that in rural areas [3].

The burden of schizophrenia is substantial, particularly for middle income countries [2, 4]. Most schizophrenia patients are supported by their main informal caregivers at home [5]. Main informal caregiver is the person who takes care and responsible for the patient without receiving any economic retribution [6]. Main informal caregivers need to confront the consequences of psychological instability of schizophrenia patients [7]. Social supports can be defined as having reliable people to assist in meeting material resource and psychosocial need [8]. Schizophrenia has been recognized as a disorder devastating for the patients’ families. The symptoms of schizophrenia may cause main informal caregivers’ mental health to deteriorate. It has been suggested that social supports help people who are in stressful situations and protect people in various pathological states [9, 10].

As potential interventions, the effect of social supports for main informal caregivers of schizophrenia patients and its application in clinical practice were demonstrated [11]. Social supports for caregivers in some western counties are more focused on a community-based multidisciplinary expert group. The content of social supports mainly includes health education, psychological intervention, mutual support group, day care, etc. The most important aspect of social supports is to help families seek out the most appropriate management approach for the patients [12].

Social supports are essential for the recovery of patients with schizophrenia. In China, social supports have been extensively studied in mental health disorders. However, little evidence is available in this area for main informal caregivers of schizophrenia patients. The potential reason is the main informal caregivers have low awareness and utilization of social supports [13].

As the capital of China, there were 79 thousand people with severe mental disorders in Beijing in 2019 [14], most of whom were schizophrenia patients. This study aims to investigate the further needs of social supports of main informal caregivers of schizophrenia patients and to analyze influencing factors on the awareness and utilization of social supports in Beijing. The results of this study could potentially act as reference for health professionals to implement appropriate and effective programs for main informal caregivers.

## Methods

### Participants and recruitment

Based on our preliminary findings, main informal caregivers of schizophrenia patients normally provided daily care for a minimum of 2 h and the duration was over 6 months. It might be because the patients in the community were in non-severe situation or stable phase.

Main informal caregivers of schizophrenia patients diagnosed by mental illness specialist hospitals, for both quantitative and qualitative investigation in this study, were approached by the inclusion criteria as follows: (1) minimum 2 h of daily care and duration over 6 months; (2) being able to answer the questions clearly and logically; and (3) willingness to participate the study.

### Data collection

#### Questionnaire survey

There are six urban districts and ten rural districts in Beijing. There were 18.65 million people in the urban districts, accounting for 86.6% of the resident population in Beijing in 2019 [15]. We carried out a cross-sectional survey in all the six urban districts from March to July 2019. A two-step sampling approach was used to minimize selection bias. First, 27 community health service centers (CHSCs) managing patients with schizophrenia were chosen from 6 urban districts by purposive sampling method. Second, all the main informal caregivers from the 27 CHSCs who met the inclusion criteria were invited to participate in the study. Total 370 caregivers were recruited. The caregivers were investigated using a questionnaire. Written informed consent was obtained from each participant prior to the investigation.

The final questionnaire comprised three parts based on literature review and preliminary findings. The first part collected the caregivers’ demographic information. The second part assessed the social supports for caregivers by the Social Support Rating Scale (SSRS) [16] with 10 items. The scale was widely used in immigrants, college students, workers, patients, patients’ caregivers, etc. for its ease of use. In the caregivers of children and adolescents with schizophrenia, the Cronbach’s α coefficient of total and subscales of SSRS were 0.818, 0.793, 0.881, and 0.836 respectively, which demonstrated high reliability and validity [17]. The 10 items are conceptually divided into three domains, including objective support (three items), subjective support (four items), and support usage (three items). Objective support reflects the degree of actual support received in the past. Subjective support reflects the perceived interpersonal network that an individual can count on. Support usage refers to the pattern of behavior that an individual utilizes when seeking social supports. The total score of the SSRS ranges from 11 to 60, with the objective support domain from 1 to 20, the subjective support domain from 7 to 28, and the support usage domain from 3 to 12. Higher scores indicate more social supports. The SSRS is a tool used to assess social supports and provides a measure of support, which is both ordinal and discrete in nature. Data of this type lend itself to analysis using ordinal logistic regression (it models the odds of being in a more sufficient support category, that is, the odds of having fair/sufficient support versus less support or sufficient support versus less/fair support). The third part collected the caregivers’ awareness and utilization of social supports.

### Individual in-depth interviews

In-depth interviews with caregivers were conducted from March to May 2019 by interviewers who received training and guidance in conducting qualitative interviews. Each in-depth interview lasted for 60 to 90 min. The interviews were structured and contained predetermined topics, in an open-ended fashion to explore caregivers’ attitudes. The topics were what kinds of supports or resources have been obtained and what further supports are needed in order to provide better care for the patients. With a mix of sex, age and experience, a total of 12 main informal caregivers were enrolled from the 370 caregivers who completed the questionnaires by stratified proportional sampling approach. Written consent was obtained from the participants with a brief introduction of the interviewers and explanation of the study. Confidentiality of data and personal information was assured to the participants.

### Data analysis

Data of questionnaires were imported into Excel 2016 and were checked by two researchers in the team. Descriptive statistics were used to describe the demographic and socioeconomic characteristics of the caregivers. Normally distributed continuous variables were described by mean value ± standard deviation (SD) and categorical variables were described by proportions. Independent t tests and analysis of variance (ANOVA) were used to analyze the mean differences in total and subscale scores by different demographic variables. Ordinal regression analysis was performed to identify potential related factors to measure the correlation between the total scores of SSRS and potential related demographic variables. All analyses were conducted using the IBM Statistical Package for Social Science Version 20.0 for Windows. All the tests are two sided, with statistical significance setting at 0.05.

All interviews were audio recorded with consents from participants. Data were analyzed using a thematic analysis. To help balance the analysis process, the team comprised professionals with different research backgrounds, including professor and graduate students of general practice, and general practitioner. Transcribed interview was independently read by research team members to identify themes by condensing and summarizing the contents. All discrepancies were discussed, and consensus reached. When no new topics were identified, data saturation was considered [18]. The point of information saturation was reached at the tenth interview in our study.

## Results

Of the 370 questionnaires administered, 363 questionnaires were returned (response rate 98.1%); 7 declined because they did not want to talk about their experiences. The mean age of the caregivers was 64.6 ± 11.5 years old. More than half of them were aged 61 years old and above. 35.5% of the caregivers received high school or higher education. Detailed socio-demographic information of the caregivers is described in Table 1.

**Table 1:** Characteristics of caregivers who completed the questionnaires (*n* = 363)

Characteristics	n (%)
Gender	
Male	154 (42.42)
Female	209 (57.58)
Age	
≤ 50 years old	38 (10.47)
51–60 years old	90 (24.79)
61–70 years old	127 (34.99)
≥ 71 years old	108 (29.75)
Ethnic group	
Han	331 (91.18)
Others	32 (8.82)
Education	
Primary school or below	36 (9.92)
Middle school degree	121 (33.33)
High school degree	129 (35.54)
College degree	51 (14.05)
Bachelor degree	22 (6.06)
Master degree or above	4 (1.10)
Religious or not	
No	328 (90.36)
Yes	35 (9.64)
Marital status	
Unmarried	11 (3.03)
Married	299 (82.37)
Divorced	17 (4.68)
Widowed	33 (9.09)
Others	3 (0.83)
Employment status	
Employed	123 (33.88)
Retired	213 (58.68)
Unemployed	26 (7.16)
Others	1 (0.28)
Relationship with patient	
Spouse	112 (30.85)
Parent	151 (41.60)
Child	31 (8.54)
Brother or sister	64 (17.63)
Other Family relative	5 (1.38)
Caring years	
≤ 5 years	7 (1.93)
6–10 years	52 (14.33)
> 10 years	302 (83.20)
Insurance	
Basic medical insurance for urban employee	221 (60.88)
Residents’ basic medical insurance	104 (28.65)
Others	38 (10.47)
Number of chronic diseases	
No	4 (1.10)
One	242 (66.67)
Two	69 (19.01)
Three and above	48 (13.22)
Hours for caring patients per day	
<6 h	173 (47.66)
6–12 h	140 (38.57)
> 12 h	50 (13.77)

Twelve informal caregivers of schizophrenia patients from 6 CHSCs in urban districts were selected for interview. Only 10 caregivers were interviewed; and the other 2 declined with no reason. Most caregivers were either spouses (6/10, *n* = 6) or children (3/10, *n* = 3) of the patients. The average age of the caregivers was 65.43 ± 11.39 years old, while the average age of the schizophrenia patients was 47.07 ± 10.45 years old. The demographics of the patients and the caregivers are described in Table 2.

**Table 2:** Characteristics of interviewed caregivers and care recipients

Items	Caregivers (*n* = 10)	Care recipient (n = 10)
Mean age	65.43 ± 11.39 years old	47.07 ± 10.45 years old
Sex		
Female	7	2
Male	3	8
Self-reported general health		
Excellent/very good	0	N/A
Good	1	N/A
Fair	3	N/A
Poor	6	N/A
Relationship		
Spouse	6	N/A
Child	3	N/A
Other family relative	1	N/A
Marital status		
Married	8	8
Widowed	2	2
Education		
Elementary school	1	3
Secondary school	5	3
Higher education	4	4
Disease status		
Stable phase	N/A	6
Relatively stable phase	N/A	2
Unstable phase	N/A	2

### Scale scores

The mean score of the scale was 27.33 ± 6.45. The distributions of total scores and subscale scores are shown in Table 3.

**Table 3:** Distribution of SSRS scores (total scores and subscale scores) of caregivers

Scale	Mean ± SD	Minimum	Maximum
SSRS	27.33 ± 6.45	12	46
Objective support	6.70 ± 2.14	1	16
Subjective support	14.56 ± 4.15	7	27
Support usage	6.07 ± 1.92	3	11

### Correlation analysis

There was no significant difference in the score by whether the caregiver was religious or not (*P* > 0.05). There were four factors positively correlated with the total SSRS score, i.e. age, education, marital status, and hours for caring patients per day (*P* < 0.05). Correlation analysis between SSRS scores (total scores and subscale scores) and general characteristics of caregivers are shown in Table S1. An additional file shows this in more detail (see Additional file 1).

### Ordinal regression analysis

We put gender, age, education, marital status, relationship with patient, main medical payment way, number of chronic diseases, and hours for caring patients per day into the regression models. The total SSRS score and the three subscale scores were dependent variables, respectively. The results revealed that insurance was a predictor of the SSRS scores. More details are shown in Additional file 1 (see Table S2).

### The caregivers’ awareness and utilization of social supports

The top three items of social support awareness were “patients could apply for disability certificate” (96.14%),“patients could obtain free psychotropic medications” (93.66%), and “patients could obtain regular medical examination once a year in the CHSCs (or stations)” (90.08%). The top three items of utilization were “patients could apply for disability certificate” (90.08%), “patients could obtain free psychotropic medications” (83.75%), and “patients could use the public transport and visit scenic spots for free” (76.58%). More details are shown in Table 4.

**Table 4:** The awareness and utilization status of social supports for caregivers

Items	Awareness rate (n%)	Utilization rate (n%)
Social security		
Patients could apply for disability certificate.	349 (96.14)	327 (90.08)
Patients could use public transport and visit scenic spots for free.	295 (81.27)	278 (76.58)
For Beijing residents, patients could apply for basic medical insurance.	189 (52.07)	126 (34.71)
For Beijing residents, patients could apply for pension subsidy.	147 (40.50)	95 (26.17)
Day care centers.	176 (48.48)	49 (13.50)
Single-child disabled family could apply for special support policies in Beijing.	54 (14.88)	21 (5.79)
Patients could apply for social insurance subsidy for disabled persons in urban areas.	46 (12.67)	21 (5.79)
Patients could participate in rehabilitation programs.	97 (26.72)	21 (5.79)
Patients could obtain financial assistance in rehabilitation programs.	54 (14.88)	11 (3.03)
Patients can participate in vocational training for disabilities.	68 (18.73)	16 (4.41)
Patients could obtain corresponding subsidies during vocational training.	47 (12.95)	8 (2.20)
Patients could be admitted to stay in a rehabilitation institution organized by the district.	58 (15.98)	15 (4.13)
Patients could obtain corresponding subsidies during their stay in a rehabilitation institution organized by the district.	29 (7.99)	11 (3.03)
Patients could apply for the reduction of individual income tax.	41 (11.29)	11 (3.03)
Patients could obtain tax incentives for Beijing disabled.	43 (11.85)	9 (2.48)
Patients could be admitted to stay in Beijing social welfare institutions for the disabled.	44 (12.12)	10 (2.75)
Patients could obtain corresponding subsidies in Beijing social welfare institutions for the disabled.	29 (7.99)	12 (3.31)
Patients could get employment support in Beijing.	58 (15.98)	3 (0.83)
Patients could get support when they starting a business in Beijing.	47 (12.95)	4 (1.10)
Financial support		
Patients could obtain disability living allowance in Beijing (100 RMB / month).	288 (79.34)	262 (72.18)
Patients could obtain Beijing residents’ home disability service (100 RMB / month).	219 (60.33)	158 (43.53)
Patients could obtain minimum living allowances for Beijing residents.	283 (77.96)	123 (33.88)
Patients could apply for basic living allowance for severely disabled families.	108 (29.75)	54 (14.88)
Patients who lived in dire poverty could get support.	112 (30.85)	49 (13.50)
Medical support		
Patients could obtain free psychotropic medications.	340 (93.66)	304 (83.75)
Patients could obtain regular medical examination once a year in the community health service center (or station).	327 (90.08)	230 (63.36)
Patients could obtain support to reduce the burden of medical expenses.	199 (54.82)	151 (41.60)
Severe patients in poverty could get psychiatric diagnosis and treatment subsidy.	109 (30.03)	76 (20.94)
Education support		
Patient’s children could obtain subsidies when be educated.	46 (12.67)	20 (5.51)
Living conditions support		
Patients or their families are supported by welfare guarantees to improve living conditions.	115 (31.68)	30 (8.26)

### Qualitative findings

Two dominant themes emerged from the insights of the caregivers: the support obtained, and further needs for social supports.

### The support obtained

Three subcategories of support obtained were identified, including medical, financial, and information support. The interviewees expressed that external financial resources like medical insurance and free medication helped to alleviate family economic pressure; information support and mutual support groups organized by community helped to alleviate psychological pressure of the caregivers. However, these types of supports were sometimes unavailable for caregivers.

*My father received 710RMB basic living allowance and 100RMB subsidy for disabled per month. A half of our house rent is paid by the government. The medications and health examinations are free for him. These have eased our financial burden to a large extent. (Caregiver 1)*

*The community health service center provides health education for the caregivers, focusing on the symptoms and medications of mental patients. (Caregiver 8)*

*I have no time to participate the mutual support group. Besides taking care of the patient, I have to work. (Caregiver 10)*

### Further needs for social supports

Three subcategories of further needs for social supports were identified, including more financial support, being respected, and affordable rehabilitation institutions. The interviewees expressed that more types of free medications should be provided by the government and coverage of healthcare insurance for patients should be broadened; a non-discriminatory environment around schizophrenia patients should be created; and rehabilitation institutions should be affordable for schizophrenia patients.

*The treatment fee for patient is too expensive. We want to get more free medical service for patients. (Caregiver 5)*

*Someone called my son idiot. This made me so sad. He should be treated fairly. (Caregiver 4)*

*The patients’ daily life was taken care of by doctors and nurses in rehabilitation institutions. It is good, but we can’t afford it. (Caregiver 10)*

## Discussion

Nine caregivers of schizophrenia patients declined to participate in this study. The stigma attached to schizophrenia might make it difficult for people to share their experience [19]. Educating the public to reduce or eliminate stigma and discrimination attached to schizophrenia is one strategy. The public health system in China should promote social awareness through mental health propaganda and education in communities, hospitals and schools to reduce or eliminate stigma and discrimination. The most important caregivers in this study were parents and spouses, and more than half of the caregivers were retirees. It is common as the caregiving responsibilities are often delegated to family members with the least economic value [20].

Available services, resources, and support for patients and their family caregivers could help to relieve the burdens on caregivers [21]. The awareness and utilization of disability certificates ranked the first in our study. Being recognized as disabled in China, people with mental disabilities are entitled to have welfare services such as medical care, rehabilitation, education, employment, and social security. Most of the schizophrenia patients (90.1%) have applied for disability certificates in this study, which makes the utilization of other social resources possible. The types of social supports for schizophrenia patients and their families in urban Beijing include medical care, social security, rehabilitation, education and training, etc. The caregivers’ awareness and utilization of resources focused more on medical care, life security, social security, and housing security. The awareness and utilization of resources such as rehabilitation and corresponding subsidies were relatively low, which were less than 10.0 and 5.0%. Although the government provided multi-channel resources for patients with schizophrenia and their families, the awareness and utilization of social supports were not optimistic. The underlying reasons might be: (1) professional rehabilitation medical workers in China were insufficient [22]; (2) burdensome application procedures and inappropriate quota allocations impaired the accessibility of support [23]; (3) stigma and discrimination from the public [24, 25], and low coverage of insurance with high treatment fees created barriers to mental health service utilization [26] (4) psychological counseling for caregivers was often considered impractical [27].

Insurance affected the caregivers’ perception of social supports in our findings. Basic medical insurance for employees is designed for the employees of urban enterprises. Residents’ basic medical insurance is designed for all urban and rural residents who are not enrolled in any insurance program. Both of the insurances are the combination of individual accounts and social pooling accounts. But the reimbursement rate of basic medical insurance for employees is higher than the rate of residents’ basic medical insurance. Inequity in health financing affects the caregivers’ usage of social supports. Improvement of the medical insurance system is a very important aspect of the current healthcare reform. Broadening the coverage of healthcare insurance and providing more accessible financial protection for patients with severe mental illness are important ways to support patients seeking medical health services [28]. The pooling fund should be increased so that it can better adjust to China’s rapidly aging population and epidemiological transitions as well as protect the insured from poverty due to illness.

Strengthening the existing primary health care system is an important measure to enhance the quality of life of schizophrenia patients and their family members. The government should allocate sufficient resources (such as rehabilitation professionals) for primary care, strengthen general practitioners’ ability to identify high-risk populations and make referral process effective [29]. There could be support groups for caregivers run by social workers or health care professionals; education or sharing sessions with physicians on typical symptoms; regular home visits and establishment of day nursing/care or respite care to meet the needs of schizophrenia patients and relieve the burden of main informal caregivers. Sufficient resources should be allocated to vocational training and supported employment for patients who were in non-severe situation or in stable phase [27].

Important aspects regarding social supports for main informal caregivers were revealed in this study and it could serve as a basis for future community-based care. But it also had some limitations. First, a cross-sectional design of the present study did not allow the determination of causal relationships. Second, the caregivers’ population in the 6 districts might not reflect the CHSCs in other parts of Beijing or other parts of China when generalizing the findings.

## Conclusions

The awareness and utilization of social supports are insufficient for main informal caregivers of schizophrenia patients. More services and better public attitudes should be considered for schizophrenia patients and their caregivers.

## Supplementary information

**Additional file 1: Table S1** Correlation analysis between SSRS scores (total scores and subscale scores) and potentially related factors. **Table S2** Ordinal regression analysis of social supports for caregivers.

## Data Availability

The datasets generated and analyzed during the current study are not publicly available to protect participant privacy, but are available from the corresponding author on reasonable request.

## Abbreviations

SSRS: Social Support Rating Scale
SD: standard deviation
CHSCs: community health service centers
