# Supplementary material for: Awareness, utilization and influencing factors of social supports for main informal caregivers of schizophrenia patients: a cross-sectional study in primary care settings in Beijing, China
Source: BMC Fam Pract. 2020 Sep 17;21:192. doi: 10.1186/s12875-020-01257-z (PMC7496203; doi:10.1186/s12875-020-01257-z)
Supplement: Supplementary file 1 — Additional file 1: Table S1 Correlation analysis between SSRS scores (total scores and subscale scores) and potentially related factors. Table S2 Ordinal regression analysis of social supports for caregivers. [file 12875_2020_1257_MOESM1_ESM.docx]

**Table S1** Correlation analysis between SSRS scores (total scores and subscale scores) and potentially related factors

| Factors | Total SSRS score | Objective support | Subjective support | Support usage |
| --- | --- | --- | --- | --- |
| Gender |  |  |  |  |
| Male | 27.05±6.27 | 6.77±2.16 | 14.47±4.03 | 5.81±1.78 |
| Female | 27.53±6.59 | 6.65±2.12 | 14.63±4.25 | 6.26±2.00 |
| t | -0.708 | 0.559 | -0.360 | **-2.237** |
| P | 0.479 | 0.577 | 0.719 | **0.026** |
| Age |  |  |  |  |
| ≤50 years old | 29.08±6.11 | 7.08±2.19 | 15.68±4.38 | 6.32±1.69 |
| 51-60 years old | 29.54±6.71 | 7.13±2.20 | 15.90±4.40 | 6.51±1.96 |
| 61-70 years old | 26.33±6.41 | 6.36±2.15 | 14.31±3.97 | 5.65±1.97 |
| ≥71 years old | 26.03±5.84 | 6.60±1.99 | 13.33±3.69 | 6.09±1.81 |
| F | **7.304** | **2.812** | **7.742** | **3.884** |
| P | **0.000** | **0.039** | **0.000** | **0.009** |
| Ethnic group |  |  |  |  |
| Han | 27.35±6.46 | 6.74±2.18 | 14.53±4.12 | 6.08±1.89 |
| Others | 27.03±6.53 | 6.31±1.60 | 14.84±4.52 | 5.88±2.24 |
| t | 0.269 | 1.075 | -0.405 | 0.590 |
| P | 0.788 | 0.283 | 0.686 | 0.556 |
| Education |  |  |  |  |
| Primary school or below | 26.17±5.45 | 6.61±2.22 | 13.47±3.08 | 6.08±2.18 |
| Middle school degree | 26.09±6.17 | 6.29±2.05 | 14.18±4.05 | 5.62±1.81 |
| High school degree | 27.95±6.92 | 6.93±2.21 | 14.80±4.42 | 6.22±1.94 |
| College degree | 28.08±6.13 | 6.82±1.94 | 14.80±4.08 | 6.45±1.82 |
| Bachelor degree | 30.73±6.43 | 7.64±2.28 | 16.23±4.54 | 6.86±1.83 |
| Master degree or above | 26.50±3.11 | 5.75±1.26 | 15.75±4.03 | 5.00±0.82 |
| F | **2.804** | **2.287** | 1.601 | **2.984** |
| P | **0.017** | **0.046** | 0.159 | **0.012** |
| Religious or not |  |  |  |  |
| No | 27.34±6.30 | 6.76±2.12 | 14.57±4.07 | 6.01±1.87 |
| Yes | 27.20±7.88 | 6.17±2.26 | 14.46±4.93 | 6.57±2.29 |
| t | 0.120 | 1.543 | 0.153 | -1.395 |
| P | 0.904 | 0.124 | 0.879 | 0.171 |
| Marital status |  |  |  |  |
| Unmarried | 27.91±5.05 | 6.73±1.74 | 14.45±3.14 | 6.73±1.95 |
| Married | 27.94±6.41 | 6.84±2.08 | 15.04±4.16 | 6.07±1.91 |
| Divorced | 23.53±8.32 | 5.53±3.17 | 12.24±4.55 | 5.76±2.33 |
| Widowed | 23.52±4.30 | 5.97±1.85 | 11.45±2.43 | 6.09±1.79 |
| Others | 27.33±4.04 | 7.67±2.08 | 14.67±2.08 | 5.00±1.73 |
| F | **5.283** | **2.753** | **7.417** | 0.662 |
| P | **0.000** | **0.028** | **0.000** | 0.619 |
| Employment status |  |  |  |  |
| Employed | 28.45±6.64 | 6.98±2.12 | 15.21±4.48 | 6.25±1.81 |
| Retired | 26.85±6.41 | 6.60±2.12 | 14.28±3.97 | 5.97±2.00 |
| Unemployed | 25.69±5.20 | 6.08±2.17 | 13.65±3.83 | 5.96±1.78 |
| Others | 34.00 | 10.00 | 17.00 | 7.00 |
| F | 2.576 | 2.455 | 1.867 | 0.677 |
| P | 0.054 | 0.063 | 0.135 | 0.567 |
| Relationship with patient |  |  |  |  |
| Spouse | 27.58±5.54 | 6.78±2.06 | 14.90±4.33 | 5.90±1.91 |
| Parent | 26.66±6.06 | 6.50±1.95 | 13.95±3.83 | 6.20±2.04 |
| Child | 26.48±6.54 | 6.84±2.31 | 13.87±4.05 | 5.77±1.56 |
| Brother or sister | 28.55±6.72 | 6.92±2.57 | 15.48±4.27 | 6.14±1.70 |
| Other Family relative | 31.40±10.24 | 7.20±2.28 | 17.60±5.90 | 6.60±3.05 |
| F | 1.665 | 0.628 | **2.718** | 0.684 |
| P | 0.158 | 0.643 | **0.030** | 0.604 |
| Caring years |  |  |  |  |
| ≤5 years | 27.14±7.38 | 7.14±2.34 | 13.71±4.57 | 6.29±1.50 |
| 6-10 years | 26.44±6.55 | 6.13±2.35 | 13.96±4.03 | 6.35±2.02 |
| >10 years | 27.50±6.45 | 6.78±2.09 | 14.69±4.18 | 6.02±1.91 |
| F | 0.591 | 2.217 | 0.824 | 0.672 |
| P | 0.554 | 0.110 | 0.439 | 0.512 |
| Insurance |  |  |  |  |
| Basic medical insurance for employees | 27.86±6.79 | 6.86±2.15 | 15.00±4.30 | 6.00±1.98 |
| Residents’ basic medical insurance | 26.88±5.67 | 6.72±2.08 | 14.04±3.82 | 6.12±1.67 |
| Others | 25.47±6.17 | 5.74±2.00 | 13.45±3.87 | 6.29±2.18 |
| F | 2.584 | **4.543** | **3.441** | 0.404 |
| P | 0.077 | **0.011** | **0.033** | 0.668 |
| Number of chronic diseases |  |  |  |  |
| None | 27.50±11.56 | 6.75±1.89 | 14.75±7.89 | 6.00±2.94 |
| One | 27.80±6.41 | 6.81±2.19 | 14.83±4.11 | 6.15±1.92 |
| Two | 27.20±6.23 | 6.68±1.94 | 14.67±4.12 | 5.86±1.94 |
| Three and above | 25.10±6.27 | 6.15±2.13 | 13.00±3.88 | 5.96±1.85 |
| F | 2.362 | 1.314 | **2.662** | 0.478 |
| P | 0.071 | 0.270 | **0.048** | 0.698 |
| Hours for caring patients per day |  |  |  |  |
| ＜6 hours | 28.03±6.73 | 6.65±2.30 | 15.15±4.33 | 6.23±2.00 |
| 6-12 hours | 27.31±6.04 | 6.89±1.80 | 14.42±3.99 | 6.00±1.68 |
| > 12 hours | 24.94±6.15 | 6.36±2.39 | 12.90±3.55 | 5.68±2.22 |
| F | **4.529** | 1.217 | **5.974** | 1.743 |
| P | **0.011** | 0.297 | **0.003** | 0.176 |

**Table S2** Ordinal regression analysis of social supports for caregivers

| Independent variable | B | Wald | *P* | 95% CI |
| --- | --- | --- | --- | --- |
| Gender |  |  |  |  |
| Male | 0.379 | 1.055 | 0.304 | -0.344~1.101 |
| Female | 0^a^ |  |  |  |
| Age |  |  |  |  |
| ≤50 years old | 0.971 | 1.780 | 0.182 | -0.455~2.397 |
| 51-60 years old | 0.993 | 2.867 | 0.090 | -0.156~2.143 |
| 61-70 years old | -0.257 | 0.278 | 0.598 | -1.210~0.697 |
| ≥71 years old | 0^a^ |  |  |  |
| Education |  |  |  |  |
| Primary school or below | 1.402 | 0.528 | 0.468 | -2.381~5.185 |
| Middle school degree | 0.220 | 0.014 | 0.906 | -3.418~3.858 |
| High school degree | 1.066 | 0.333 | 0.564 | -2.558~4.690 |
| College degree | 0.891 | 0.223 | 0.637 | -2.810~4.592 |
| Bachelor degree | 2.478 | 1.638 | 0.201 | -1.317~6.272 |
| Master degree or above | 0^a^ |  |  |  |
| Marital status |  |  |  |  |
| Married | -1.526 | 0.481 | 0.488 | -5.837~2.785 |
| Divorced | -0.103 | 0.003 | 0.959 | -3.986~3.781 |
| Widowed | -1.843 | 0.775 | 0.379 | -5.945~2.260 |
| Others | -0.676 | 0.110 | 0.741 | -4.682~3.329 |
| Unmarried | 0^a^ |  |  |  |
| Relationship with patient |  |  |  |  |
| Spouse | -1.812 | 2.138 | 0.144 | -4.242~0.617 |
| Parent | -1.999 | 2.545 | 0.111 | -4.456~0.457 |
| Child | -2.395 | 3.104 | 0.078 | -5.059~0.269 |
| Brother or sister | -0.955 | 0.610 | 0.435 | -3.351~1.442 |
| Other Family relative | 0^a^ |  |  |  |
| Insurance |  |  |  |  |
| Basic medical insurance for employees | 1.310 | 5.803 | **0.016** | 0.244~2.375 |
| Residents’ basic medical insurance | 1.228 | 4.388 | **0.036** | 0.079~2.377 |
| Others | 0^a^ |  |  |  |
| Number of chronic diseases |  |  |  |  |
| No | -0.080 | 0.002 | 0.964 | -3.548~3.570 |
| One | 0.523 | 1.081 | 0.298 | -0.463~1.547 |
| Two | 0.383 | .434 | 0.510 | -0.757~1.524 |
| Three and above | 0^a^ |  |  |  |
| Hours for caring patients per day |  |  |  |  |
| ＜6 hours | 0.196 | 0.146 | 0.703 | -0.811~1.203 |
| 6-12 hours | 0.261 | 0.253 | 0.615 | -0.757~1.280 |
| >12 hours | 0^a^ |  |  |  |

CI: confidence interval
